# Neural mechanisms of predicting individual preferences based on group membership

**DOI:** 10.1093/scan/nsaa136

**Published:** 2020-10-07

**Authors:** Suhas Vijayakumar, Egbert Hartstra, Rogier B Mars, Harold Bekkering

**Affiliations:** Donders Institute for Brain, Cognition and Behaviour, Radboud University Nijmegen, HR, Nijmegen, The Netherlands; Donders Institute for Brain, Cognition and Behaviour, Radboud University Nijmegen, HR, Nijmegen, The Netherlands; Donders Institute for Brain, Cognition and Behaviour, Radboud University Nijmegen, HR, Nijmegen, The Netherlands; Wellcome Centre for Integrative Neuroimaging, Centre for Functional MRI of the Brain (FMRIB), Nuffield Department of Clinical Neurosciences, John Radcliffe Hospital, University of Oxford, Oxford, United Kingdom; Donders Institute for Brain, Cognition and Behaviour, Radboud University Nijmegen, HR, Nijmegen, The Netherlands

**Keywords:** mentalizing, fMRI, theory of mind, social learning

## Abstract

Successful social interaction requires humans to predict others’ behavior. To do so, internal models of others are generated based on previous observations. When predicting others’ preferences for objects, for example, observations are made at an individual level (5-year-old Rosie often chooses a pencil) or at a group level (kids often choose pencils). But previous research has focused either on already established group knowledge, i.e. stereotypes, or on the neural correlates of predicting traits and preferences of individuals. We identified the neural mechanisms underlying predicting individual behavior based on learned group knowledge using fMRI. We show that applying learned group knowledge hinges on both a network of regions commonly referred to as the mentalizing network, and a network of regions implicated in representing social knowledge. Additionally, we provide evidence for the presence of a gradient in the posterior temporal cortex and the medial frontal cortex, catering to different functions while applying learned group knowledge. This process is characterized by an increased connectivity between medial prefrontal cortex and other mentalizing network regions and increased connectivity between anterior temporal lobe and other social knowledge regions. Our study provides insights into the neural mechanisms underlying the application of learned group knowledge.

## Introduction

Humans live in a complex social world in which we interact with a vast number of individuals. Successful social interaction requires humans not only to passively respond to the behavior of others, but also to predict their behavior. To do so, internal models of others need to be generated based on previous experiences (Clark, 2013; Koster-Hale and Saxe, 2013; Diaconescu *et al.*, 2014). These models should capture the beliefs, mental states and traits causing the behavior of others (Gallagher and Frith, 2003; Saxe and Kanwisher, 2003; Frith and Frith, 2006; Koster-Hale and Saxe, 2013). However, these causes are highly variable across the many individuals that we interact with, making it difficult to produce accurate predictions tailored to each individual (Waytz *et al.*, 2010; Rhodes, 2013). One way to reduce this uncertainty is by using knowledge based on the group membership of an individual (hereafter referred to as group knowledge) that captures the shared features of its members (Lieberman, 2007; Mitchell *et al.*, 2009; Amodio, 2014).

The tendency to cluster our social environment into discrete categories is a process that is developed as early as infancy (Kinzler *et al.*, 2010; Rhodes, 2013). To date, research into the neural basis of social information processing has mainly focused on either the mechanisms underlying the inferences of beliefs and mental states of another individual (Gallagher and Frith, 2003; Saxe and Kanwisher, 2003; Saxe *et al.*, 2004a; Frith and Frith, 2006) or on how already established knowledge about groups (e.g. stereotypes) bias our judgment of individuals (Greenwald *et al.*, 2002; Lieberman, 2007; Mitchell *et al.*, 2009; Contreras *et al.*, 2012; Amodio, 2014). Inferring the mental states of others has been shown to involve the frontal–temporal network that include the medial prefrontal cortex (mPFC), superior temporal sulcus (STS) and temporal parietal junction (TPJ) (Saxe and Kanwisher, 2003; Frith and Frith, 2006; Koster-Hale and Saxe, 2013). Of these regions, the mPFC is not only relevant for identifying self within a group (Morrison *et al.*, 2012), but also for situating oneself in a newly formed group (Molenberghs and Morrison, 2014). It is considered essential for the application of group knowledge, that is, when one needs to make judgments about individual traits based on the group membership of a particular individual (Zahn *et al.*, 2007; Amodio, 2014), and has been found to play a vital role when predicting others’ behavior (Amodio and Frith, 2006; Frith and Frith, 2006; Amodio, 2014). Besides this core ‘social network’, the retrieval of long-term semantic social knowledge particularly recruits the anterior temporal lobe (aTL) (Skipper *et al.*, 2011), which has been implicated in representing and retrieving social knowledge (Olson *et al.*, 2013), stereotype representation (Contreras *et al.*, 2012) and in the retrieval of attributes that describe people, but not objects (Zahn *et al.*, 2007; Amodio, 2014). Furthermore, aTL has been associated with the acquisition of prejudice regarding newly formed social groups (Spiers *et al.*, 2017).

Based on previous research discussed above, we hypothesized that forming internal models of an individual’s behavior based on group knowledge relies both on the mPFC and aTL. More specifically, we hypothesize that the mPFC is particularly involved in the formation of internal models capturing others’ behavior, while the aTL is essential for acquiring and representing group knowledge, in line with its role in representing semantic social knowledge. To identify the neural mechanisms underlying acquisition of group knowledge and predicting other individuals’ behavior based on learned group knowledge, we devised a novel social learning functional magnetic resonance imaging (fMRI) paradigm. In this task, participants had to infer object preferences of virtual agents. However, these object preferences needed to be learned over the course of the experiment and were related to the specific social group that the virtual agents belonged to. Crucially, in order to be successful in this task, participants had to form internal models of these agent-specific preferences based on group knowledge that was learned over the course of the experiment. Furthermore, to test whether the aTL is especially involved in representing already learned group knowledge, half of the object-preference contingencies were trained prior to the fMRI session. We hypothesized that during these trials aTL involvement would be the highest.

## Methods

### Participants

Twenty-seven right-handed individuals (15 female, *M* age = 24.52 years, s.d. = 3.79) were recruited to take part in the study through a university-wide online registration system—Radboud Research Participation System (SONA). Participants reported to be healthy and had no history of neurological disorders. They had normal or corrected-to-normal vision at the time of the experiment. All participants gave their written informed consent according to the institutional guidelines of the local ethics committee (CMO region Arnhem-Nijmegen, The Netherlands) and received financial compensation of €25.50 for their participation.

Data from three participants were excluded because they were unable to complete the experiment. Data from two more participants were excluded due to high error scores (65.91% and 62.24%), determined as not significantly different from chance-level performance by binomial test, which was an a priori set criteria for exclusion (see Supplementary text; ‘participant exclusion criteria’). The final dataset consisted of 22 participants (12 female, *M* age = 24.68 years, s.d. = 3.52) whose data were included in further analyses.

### Task

**Fig. 1.**
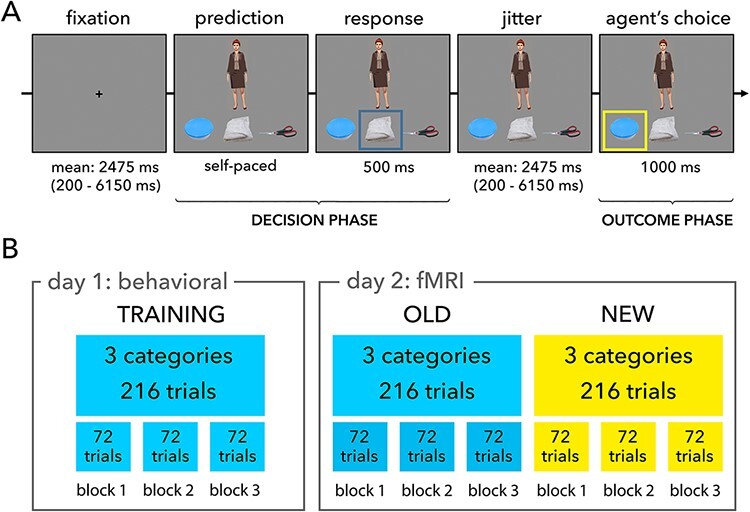
A. Task overview. Each trial started with a fixation cross after which the decision phase was presented. This phase consisted of the presentation of an agent with three objects placed underneath the agent. A blue box appeared around the object chosen by the participant marking the end of the decision phase. Next the blue box disappeared and the agent along with the three objects remained visible on the screen. Thereafter the outcome phase was presented depicting the choice of the agent via the presentation of a yellow square around the chosen object. Note: The agent depicted in this figure closely resembles one of the agents used in the experiment and is not from the actual stimulus set. B. Schematic overview of trial distribution over blocks and conditions for the training session (day 1: Behavioral) and fMRI session (day 2: fMRI). Blue blocks represent trials used in both sessions (OLD condition) and yellow blocks represent trials only used during the fMRI session (NEW condition).

Each condition of the experiment consisted of three agent categories and three object categories. On each trial, a virtual agent was presented along with images of three objects below them. The task was to predict which of the three objects the agent would choose (see Figure 1A, for task overview). The virtual agents could be grouped into agent categories based on the subculture of their clothing style (artistic black, summer dress, casual, etc.). Similarly, the objects could be grouped into specific object categories (keys, tupperware, cups, etc.). Each agent category had a specific preference for an object category that was manipulated in a probabilistic fashion. Agents from each agent category made an appearance in a total of 72 trials. Within those trials, agents chose an object from the most preferred category 45 times (62.5%), an object from less preferred category 18 times (25%) and an object from the least preferred category 9 times (12.5%). It is important to note that there was no relation in terms of preference between any specific individual agent and any specific object. No agent chose the same object twice. The association and hierarchy of preferences thus existed at the level of agent categories and not at the level of individuals.

Half of the object-preference contingencies were trained prior to the experimental session. Consequently, the neuroimaging task consisted of agent categories whose preferences were already learned and needed to be retrieved and novel agent categories whose preferences needed to be learned over the course of the experimental session (see below for more details).

### Stimulus material

Stimulus material consisted of 108 virtual agents that could be grouped into 9 agent categories based on subculture of their clothing style, with 12 agents in each social group. The virtual agents were designed using SIMS 4 (Electronic Arts Inc., 2014), and their group consistency was verified by means of a separate pilot experiment (for more details, see Supplementary material, section ‘stimulus validation’). All virtual agents were white females and were portrayed in the same posture, to avoid possible confounds arising from such differences. An equal number of object categories were created with 12 objects in each category by means of photos of 108 objects (see supplementary material, section “stimulus material”. Supplementary Figure S2 contains all of the object images used in the experiment), so that each social group could be matched to have a preference for an object category (see Table 1 for an overview of the agent categories and object categories used in the experiment and Supplementary Table S3 for agent categories and their respective object category preferences used in the experiment for each participant). The objects used were neutral and care was taken to avoid obvious natural preference with the agent categories that they were presented with. For example, trials with agents belonging to sportswear group were never presented with towels as one of the object categories to avoid any preconceived notion of real-world preferences.

**Table 1.** List of agent categories and object categories

Agent categories	Object categories
Office-wear	Towels
Artistic black	Keys
Sporty	Cups
Casual	Plates
Summer dress	Marker pens
Long dresses	Scissors
Short pants	Bowls
Tattooed	Cleaning brushes
Punk	Tupperware

Agents from artistic black category and tattoo category were not presented in the same block due to similarity in the color of their clothes.

### Design and procedure

The experiment was programmed using Presentation software version 17 (Neurobehavioral Systems, Inc.) and was conducted on two consecutive days. On day 1, participants took part in a behavioral training phase involving a 24 trials practice block, followed by an experimental session of 3 blocks, each containing 72 trials. Stimuli were presented on an LCD monitor and participant responses were recorded using a keyboard. Day 2 consisted of the fMRI session with a 24 trials practice block to accustom participants to the scanner environment; neuroimaging data were not recorded during this period. The practice block was followed by two experimental sessions. One session consisted of the already learned agent category—object category preference contingencies from day 1 training phase (hereafter referred to as OLD condition) and the other consisted of novel agent categories and their preferences for object categories that had to be learned over the course of the session (NEW condition). So, each participant encountered 3 agent-object category pairs on 2 days (OLD condition), 3 category pairs were encountered only once, during fMRI session (NEW condition), and the remaining 3 category pairs were used for practice on both days. The presentation order of the NEW and OLD condition was counterbalanced across participants to account for differences caused by the presentation order. Each condition consisted of 3 blocks containing 72 trials each. After each block, participants could take a break before proceeding to the next block. With 3 blocks in each condition, the experiment followed a 2 × 3 factorial design, with condition (NEW, OLD) × block (BLOCK1, BLOCK2, BLOCK3) as factors (see Figure 1B for a schematic overview of experimental setup).

Stimuli were rear-projected onto a screen with grey background (Eiki LCD projector, 60 Hz refresh rate, 1024 × 768 display resolution), which was visible to the participants through a mirror attached to the head coil. Participants gave their responses via a button box, using their index, middle and ring finger representing each of the three objects on screen. On both the days, agent-object categories used during the practice block were not used during experiment blocks. Behavioral training session lasted for ~50 minutes and fMRI session took ~90 minutes, including providing instructions and the practice block.

### Trial structure

Each trial began with a fixation cross (Figure 1A) that lasted for a time determined by a pseudo-logarithmic distribution (*M* = 2475 ms, range = 200–6150 ms; see Vergauwe *et al.* (2015) or De Baene *et al.* (2012) for a similar procedure). Using steps of 350 ms, 50% of the trials used a jitter ranging from 200 to 1950 ms, 33.33% of the trials used a jitter ranging from 2300 to 4050 ms and 16.67% of the trails used a jitter time ranging from 4400 to 6150 ms Following this, an agent was presented along with three objects (note that the agent shown in Figure 1A closely resembles one of the agents used in the experiment). This was followed by the ‘Decision phase’, where participants had to make a decision about the preferred object of the agent, based on the preference of the social group that this agent belonged to. Participants were allowed to take their time and respond in the form of a button press. Participants’ response was confirmed to them by the presentation of a blue square around their object of choice. The blue square disappeared after 500 ms and a screen depicting the agent and the three objects was presented for a variable jitter time that followed earlier mentioned pseudo-logarithmic distribution. After this jittered delay, the object chosen by the agent was revealed to the participant by means of a yellow square around the object for 1 s, this constituted the ‘Outcome phase’. The next trial started immediately thereafter.

### Distribution of trials

Both the NEW and OLD conditions consisted of three blocks, with a set of three agent-object categories in each condition. The 62.5% of first preference, 25% of second preference and 12.5% of third preference object selection was maintained throughout. Among a total of 216 trials, first preference object was chosen 135 times, second preference object 54 times and third preference object 27 times. This distribution was also maintained at a block level of 72 trials (first preference = 45 trials, second preference = 18 trials, third preference = 9 trials), as well as in sub-blocks of 24 trials within each trial-block (first preference = 15 trials, second preference = 6 trials, third preference = 3 trials). Agents from all three agent categories were presented equal number of times within each of these sub-blocks. An additional constraint on the sequence of stimuli presentation was that first preference object was chosen no >4 times in a row. Also, the number of times an agent picks a preferred or non-preferred object was restricted. Every agent chose an object from the most preferred category at least 3 times, and up to 4 times (see Supplementary Table S1 for an example). Lastly, associations between social group and object categories were pseudo-randomly assigned for each participant avoiding any obvious real-world association between them.

### Behavioral analysis

Participant responses during fMRI session were excluded from the dataset if their reaction times (RTs) were <300 ms, or if RTs were >3 s.d. away from the mean. Participants were judged based on the number of trials in which they correctly predicted the first preference object of the social group. Although the task was to predict what the agent would choose on each trial, participants could only succeed by consistently predicting the object of first preference since that had the highest chance of selection. So, participant response on any given trial was considered ‘correct’ as long as they selected the most preferred object category of the social group irrespective of what the agent chose on that particular trial. To verify that a participant’s performance was significantly above chance level during NEW trials of day 2, we used one-directional binomial test. If a participant’s performance was above chance level, their data were included in further analyses.

In order to gauge the efficiency of the participant predictions we computed the efficiency index of participants for each block as (Woltz and Was, 2006; Machizawa and Driver, 2011),

$$\begin{equation*}Efficiency\,\,index = {{probability\,\,of\,\,a\,\,correct\,\,response} \over {mean\,\,RT}}\end{equation*}$$

Efficiency index scores were computed separately for each block and condition. We used a 2 × 3 repeated-measure analysis of variance (ANOVA) to determine the effects of condition (NEW, OLD) and block (BLOCK1, BLOCK2, BLOCK3) on efficiency index scores. Bonferroni corrected post hoc tests were further conducted upon finding significant main effects or effects of interaction.

While the binomial test was performed using a MATLAB script- (Binomial test: http://www.mathworks.com/matlabcentral/fileexchange/24813-mybinomtest-s-n-p-sided-), all other behavioral analyses were carried out using version 22 of the Statistical Package for the Social Science (SPSS 22; IBM Corporation, Armonk, NY, USA).

### fMRI methods

Participants were scanned in a Siemens 1.5 T magnetic resonance imaging (MRI) scanner as they lay in head-first supine position with their head movement restricted using foam cushions and a tape running along their forehead. Following a localizer sequence, 176 high-resolution anatomical images were acquired using T1-weighted MPRAGE sequence (TR = 2250 ms, TE = 2.95 ms, image matrix = 256 × 256, FOV = 256 mm, flip angle = 15^0^, slice thickness = 1 mm, voxel size = 1.0 × 1.0 × 1.0 mm). A T2*-weighted multi-echo echo-planar imaging (EPI) sequence was used to acquire BOLD-sensitive functional images during task performance (TR = 2470 ms, TE1 = 7.0 ms, TE2 = 26.3 ms, TE3 = 36 ms, TE4 = 45 ms, TE5 = 54 ms, image matrix = 64 × 64, FOV = 224 mm, flip angle = 80^0^, slice thickness = 3.0 mm, distance factor = 17%, voxel size = 3.5 × 3.5 × 3.0 mm, 31 axial slices). Number of images acquired in each run was unequal as participants took varying time to complete each block of the experiment.

The functional images were preprocessed and analyzed using SPM8 (Statistical Parametric Mapping version 8 http://www.fil.ion.ucl.ac.uk/spm/software/spm8/, from the Wellcome Department of Neurology, London, UK), implemented in MATLAB 2012 (Mathworks Inc., Sherborn, MA, USA). To allow for magnetization to reach its equilibrium, the first five scans of each EPI series were excluded from the analysis. During preprocessing, all functional images were first spatially realigned using rigid body transformation and a mean image of all functional scans of each participant was created. Functional scans were corrected for differences in slice time using first slice as the reference. Structural image of each participant was co-registered with their mean functional image and all functional images were normalized to the Montreal Neurological Institute (Montreal, Quebec, Canada) T1 template. The images were then spatially smoothed using a 9 mm full width at half maximum Gaussian filter and further statistical analyses were performed on each participant’s data using the general linear model (GLM) in SPM8.

### fMRI analyses

To identify brain regions underlying the learning of group knowledge when predicting other individuals’ behavior, a GLM was fitted to the fMRI data for each participant. The main aim of the current study was to investigate which neural mechanisms underlie learning to predict the behavior of others based on group knowledge. To this end, we focused our analyses on the ‘decision phase’, that is, on the moment that participants needed to predict which object the agent was most likely to choose. Therefore, the GLM included six regressors of interest that used the onset time of the decision phase with a duration that was equal to participant’s RT for that particular trial. These regressors followed the 2 × 3 design of the experiment and as such distinguished between the NEW and OLD condition as well as their corresponding blocks (e.g. BLOCK1, BLOCK2, BLOCK3) for a total of six regressors. We also modeled the ‘outcome phase’ using two regressors that corresponded to a prediction match (participant’s prediction was the same as the object choice of the virtual agent) or prediction error (there was a mismatch between the participant’s prediction and the choice of the virtual agent), using the onset of the feedback presentation as onset times, having a duration of 1 s. Separate regressors were included for each condition and blocks. So, 2 prediction outcomes (MATCH, ERROR) x 2 condition (OLD, NEW) x 3 blocks (BLOCK1, BLOCK2, BLOCK3) resulting in 12 regressors for the outcome phase. In total, this resulted in 18 task regressors. For these 18 task regressors, both a canonical hemodynamic response function (HRF) and the first derivative were modeled. Following Friston *et al.* (1996), head-movement effects were accounted for by including a Volterra expansion of the six rigid-body motion parameters as nuisance covariates, which consisted of linear and quadratic effects of the six realignment parameters belonging to each volume. It also included spin-history effects as linear and quadratic effects of motion parameters in the previous volume, giving a total of 36 motion regressors (Lund *et al.*, 2005). To remove low-frequency signal drifts, a 128 s high-pass filter was applied.

Contrast images were computed for the six decision phase regressors containing the canonical HRF for each participant. Thereafter, individual contrast images were submitted to a 2 × 3 full factorial second level group analyses, with condition (NEW, OLD) and block (BLOCK1, BLOCK2, BLOCK3) as factors, treating participants as random effects. At the second level whole-brain one-sample *t*-tests contrasts were computed. The resulting activation maps were tested for significance at a voxel level threshold of *P* < 0.001 (uncorrected) while correcting for multiple comparisons using a family-wise error (FWE) cluster-corrected probability of *p* < 0.05.

### Functional connectivity analysis

To further understand the nature of interactions between brain regions involved in learning to predict others’ behavior based on group knowledge, a generalized psychophysiological interaction (gPPI; http://www.nitrc.org/projects/gppi) analysis was performed. gPPI when compared with the standard PPI has been shown to be more sensitive toward true positive results (McLaren *et al.*, 2012). In addition, it is more flexible in terms of the statistical models one can use as it can cover the whole experimental design without the restriction that each condition has to occur in all scan runs (McLaren *et al.*, 2012). This makes it suitable for the current design as learning occurs over blocks. Considering our hypotheses that both mPFC and aTL play a crucial role in predicting other individual’s behavior based on learned group knowledge, we used these areas as seed regions. Both seed regions were defined as spheres (radius, Ø = 10 mm) and were centered around peak voxels of activation found in the whole-brain contrasts. While mPFC showed increased activation in both contrasts, we were interested in studying its functional connectivity while predicting individual preferences based on group membership. So, for mPFC seed, the sphere was centered around MNI coordinates (−6, 50, 7), reflecting the voxel of peak activation found in the ‘BLOCK3 > BLOCK1’ whole-brain contrast. As we expected mPFC to be involved in utilizing group knowledge that is represented elsewhere, the aTL (−42 14–29) MNI coordinates were taken from the CONDITION × BLOCK whole-brain interaction contrast.

After defining the seed regions, their time courses were extracted, and a connectivity analysis was conducted using gPPI toolbox with its default configuration of parameters. A first-level analysis was conducted for the two seed regions separately, adding a PPI regressor to the previously described GLM. Our main interest was to capture changes in functional connectivity linked to increased learning of predictions based on group knowledge, which should be reflected in changes in connectivity during BLOCK3 trials as compared to BLOCK1 trials. As such *t*-contrast images we created contrasting the gPPI BLOCK3 regressor with the gPPI BLOCK1 regressor. A group-level analysis was then performed by entering participant-specific *t*-contrast images into a one-sample *t*-test. Resulting activation maps were tested for significance at a voxel level threshold of *P* < 0.005 (uncorrected) while correcting for multiple comparisons using an FWE cluster-corrected probability of *P* < 0.05.

## Results

### Behavioral results

To measure participants’ improvement in learning to predict others’ behavior based on group knowledge we computed the efficiency index for each condition (NEW, OLD) and block (BLOCK1, BLOCK2, BLOCK3) separately. Behavioral results in terms of mean RTs and accuracy are reported in Supplementary material for additional insight (Supplementary Figure S3). We observed both an increase in accuracy and a decrease in RTs. The latter could indicate that participants become more certain about their predictions as the task progressed. Efficiency index was defined based on previous literature (Woltz and Was, 2006; Machizawa and Driver, 2011) as,

$$\begin{equation*}Efficiency\,\,index = {{probability\,\,of\,\,a\,\,correct\,\,response} \over {mean\,\,RT}}\end{equation*}$$

All behavioral analyses were performed on the fMRI session data.

**Fig. 2.**
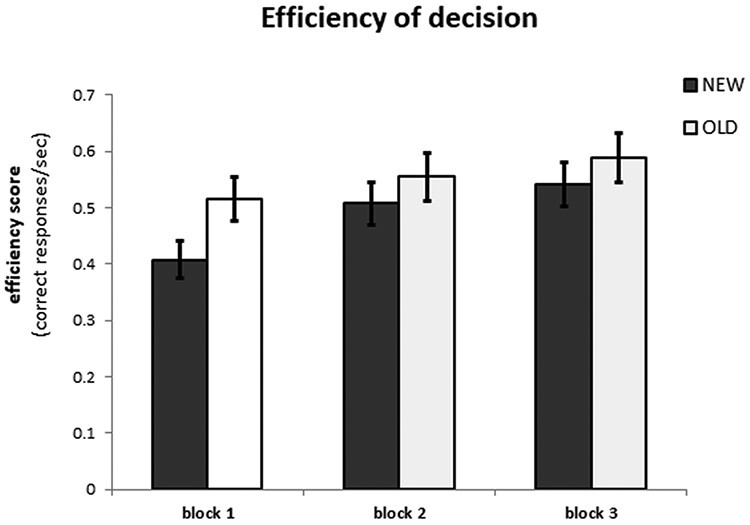
Bar plot of performance during the fMRI session represented in the efficiency scores (proportion of correct responses divided by mean response time in seconds, plotted on the y-axis) for each block (x-axis) and condition (NEW condition: grey bars, OLD condition: white bars) separately. Error bars represent standard error of the means.

The repeated-measure ANOVA with factors CONDITION (NEW, OLD) × BLOCK (BLOCK1, BLOCK2, BLOCK3) performed on efficiency index scores revealed both a main effect of CONDITION, *F*(1, 21) = 6.92, *P < *0.05, *η^2^* = 0.25 and BLOCK, *F*(2, 42) = 37.10, *P < *0.001, *η^2^* = 0.64. Efficiency scores were higher during OLD trials (*M *= 0.55, s.d.* *= 0.04) as compared to NEW trials (*M *= 0.49, s.d.* *= 0.035). Furthermore, pairwise comparisons revealed that efficiency scores increased as learning progressed; the differences between all three blocks were significant (all *P’s *< 0.05; BLOCK1: *M *= 0.46, s.d.* *= 0.034, BLOCK2: *M *= 0.53, s.d.* *= 0.037 and BLOCK3: *M *= 0.57, s.d.* *= 0.037). The interaction effect of CONDITION × BLOCK was also significant, *F*(2, 42) = 3.71, *P*< 0.05, *η^2^* = 0.15. To further characterize this interaction effect, two follow-up repeated-measure ANOVAs were conducted with BLOCK (BLOCK1, BLOCK2, BLOCK3) as a factor (see Figure 2).

For the NEW trials there was a main effect of BLOCK, *F*(2,42) =  30.30, *P < *0.001, *η^2^* = 0.59. Pairwise comparisons revealed that the efficiency scores during BLOCK1 (*M* = 0.41 s.d.* *= 0.033) were significantly lower than during BLOCK2 (*M* = 0.51 s.d.* *= 0.038) and BLOCK3 (*M* = 0.54 s.d.* *= 0.039), all *P*’s* *< 0.001. The difference between BLOCK2 and BLOCK3 failed to reach significance, *P *> 0.1. For OLD trials this analysis resulted in a main effect of BLOCK, *F*(2,42) = 8.57, *P *=* *0.001, *η^2^* = 0.29. Pairwise comparisons revealed that efficiency scores were only significantly lower in BLOCK1 (*M* = 0.52 s.d.* *= 0.039) than in BLOCK3 (*M* = 0.59 s.d.* *= 0.043), *P *< 0.001. BLOCK2 scores (*M* = 0.56 s.d.* *= 0.043) were not significantly different from BLOCK1, *P *> 0.1, or BLOCK3 scores, *P* > 0.2.

In sum, the behavioral results show that irrespective of whether one was making predictions when acquiring group knowledge (NEW condition) or making predictions based on already learned group knowledge (OLD condition), there was an increase in prediction performance as learning progressed, particularly when tested for difference in performance between BLOCK1 and BLOCK3.

### fMRI results

#### Learning to predict others’ behavior based on group knowledge.

All fMRI analyses focus on the ‘decision phase’, that is, at the moment that participants needed to predict which object the agent was most likely to choose based on group membership.

In order to investigate whether there were regions that showed a general increase when predictions were based on already learned group knowledge, OLD trials were contrasted with NEW trials. It was during OLD trials that already learned group knowledge could be retrieved. The reversed contrast was also conducted to investigate whether any region showed specific sensitivity to NEW trials. Both contrasts did not reveal any significant clusters of activation, showing that there was no general increase in activation for OLD or NEW trials.

**Fig. 3.**
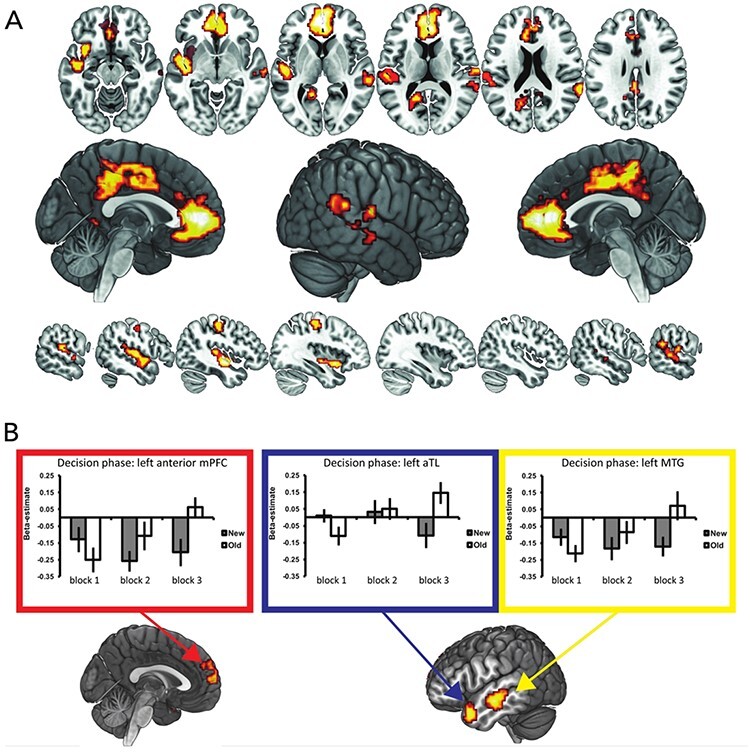
A. Whole-brain activation map stemming from the overall time effect contrast (BLOCK3 > BLOCK1). B. Whole-brain activation map stemming from the interaction contrast [OLD BLOCK3—OLD BLOCK1] > [NEW BLOCK3—NEW BLOCK1]. Bar graphs represent beta-estimates (y-axis) for each block (x-axis) and condition (NEW condition grey bars, OLD condition white bars) are shown separately for the anterior mPFC (red box), left aTL (blue box) and left MTG (yellow box).

As demonstrated by the behavioral data, predictions made during both OLD and NEW conditions showed increased improvement as the task progressed, especially between BLOCK1 and BLOCK3 trials. To investigate whether there were brain regions that showed increased involvement as learning progressed over time, we contrasted BLOCK3 trials with BLOCK1 trials (‘overall time effect contrast’). This contrast showed an increase in activation in several regions including the ventral part of mPFC including the medial frontal pole area FPm and area 32pl (Neubert *et al.*, 2015), right TPJ extending into STS, left superior temporal gyrus, precuneus, dorsal mid-cingulate and posterior cingulate cortex (Beckmann *et al.*, 2009), primary somatosensory cortex and ventral parietal occipital sulcus (see Figure 3A and Table 2 for an overview of the anatomical labels and their MNI coordinates, and see Supplementary Figure S4 for beta estimates extracted from spherical region of interest of 10 mm radius, drawn at activation peaks of these regions). To investigate whether there were regions that showed a decline in activation over the course of learning the reversed contrast (BLOCK1 > BLOCK3) was computed, which did not yield any significant clusters of activation.

**Table 2.** Overview results ‘overall time effect contrast’ (BLOCK3 > BLOCK1)

Anatomical location	Voxels	z value	MNI coordinates (x, y, z)	Laterality
Paracingulate gyrus	936	5.54	−6 50 7	Left
Anterior cingulate gyrus		5.18	0 41 7	Middle
Rostral gyrus		4.79	−12 44 −5	Left
Superior temporal gyrus	409	5.04	−42 −10 −8	Left
Insula		4.59	−36 8 −11	Left
Superior temporal gyrus		4.44	−51 −19 4	Left
Para-central lobule	686	4.50	15 −34 49	Right
Precuneus		4.31	6 −37 52	Right
Cingulate gyrus		4.10	9 −31 43	Right
Post-central gyrus	130	4.35	−42 −22 52	Left
Pre-central gyrus		4.18	−36 −25 58	Left
Pre-central gyrus		3.31	−21 −25 58	Left
Superior temporal gyrus	167	3.91	66 −46 22	Right
Superior temporal gyrus		3.79	63 −37 16	Right
Superior temporal gyrus		3.77	66 −19 10	Right
Ventral parietal–occipital sulcus	114	3.86	−18 −49 7	Left
Ventral parietal–occipital sulcus		3.83	−24 −64 13	Left
Ventral parietal–occipital sulcus		3.65	−12 −55 13	Left

Behaviorally, predictions during OLD trials showed higher efficiency scores as compared to predictions made during NEW trials. During the OLD trials, predictions still improved as time progressed. This suggests that learning group knowledge reaches its peak during OLD BLOCK3 trials. In order to test if there were brain regions that specifically showed a peak in activation over time in the OLD condition as compared to the NEW condition we computed the ‘interaction contrast’ of [OLD BLOCK3—OLD BLOCK1]-[NEW BLOCK3—NEW BLOCK1]. This revealed anterior parts of mPFC, at a location more dorsal than in the BLOCK3 > BLOCK1 main effect overlapping with the medial frontal pole but also extending into area 9 m (Neubert *et al.*, 2015), the left aTL, and bilateral middle temporal gyrus (MTG) (Figure 3B; Table 3). In order to verify that this interaction effect was caused by the increased involvement of these areas specifically during OLD BLOCK3 trials, we extracted the beta-estimates for these regions by drawing a sphere (radius = 10 mm) around their peak activation. This confirmed that all the areas identified by the interaction contrast especially showed increased involvement during OLD BLOCK3 trials (Figure 3B bar graphs). These results demonstrated the involvement of regions of the mentalizing network and aTL in predicting individual preferences based on group membership and differ from regions involved in the trial-by-trial performance of the task that are discussed in the Supplementary material (under section “parametric modulation results”).

**Table 3.** Overview results whole-brain ‘interaction contrast’ (OLD BLOCK3 > OLD BLOCK1) > (NEW BLOCK3 > NEW BLOCK1)

Anatomical location	Voxels	z value	MNI coordinates (x, y, z)	Laterality
Middle temporal gyrus	169	4.61	−57 −13 −14	Left
Middle temporal gyrus		3.93	−57 −7 −26	Left
Middle temporal gyrus		3.70	−48 −13 −11	Left
Temporal pole	75	4.54	−42 14 −29	Left
Temporal pole		3.42	−51 14 −17	Left
Superior temporal sulcus	109	4.53	48 −16 −14	Right
Superior temporal sulcus		3.75	60 −10 −11	Right
Medial frontal pole	247	4.20	−9 65 7	Left
Paracingulate sulcus		3.83	6 53 25	Right
Anterior Medial superior frontal gyrus		3.79	9 62 28	Right

##### Changes in functional connectivity underlying retrieving learned group knowledge.

**Fig. 4.**
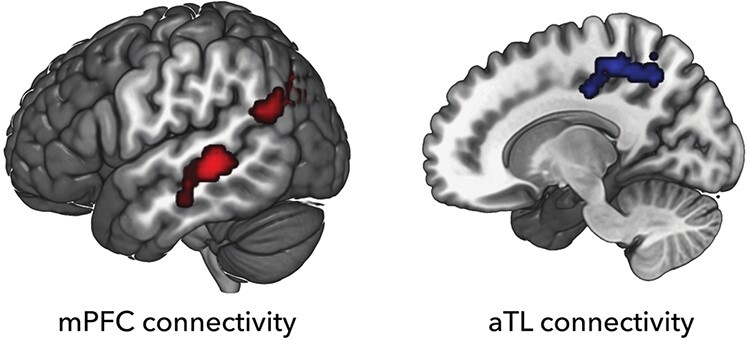
Whole-brain activation map showing increased functional connectivity during BLOCK3 trials as compared to BLOCK1 trials. Regions in red showed increased connectivity with the mPFC seed region. Regions in blue showed increased connectivity with the aTL seed region.

Since we were especially interested in the role of mPFC and aTL in predicting others’ behavior based on group knowledge, we performed gPPI analysis using these two regions as seeds. More specifically, we wanted to gauge changes in functional connectivity that are linked to changes in connectivity during BLOCK3 trials as compared to BLOCK1 trials, irrespective of OLD or NEW conditions, as it was during BLOCK3 trials that predictions could be based on learned group knowledge. The mPFC showed increased functional connectivity with the left temporo-parietal cortex in an area overlapping with the posterior TPJ (Mars *et al.*, 2012b) and left STS, while the aTL showed increased functional connectivity with the para-central lobule and parts of the precuneus (Figure 4, Tables 4 and 5).

**Table 4.** Overview results mPFC functional connectivity analysis

Anatomical location	Voxels	z value	MNI coordinates (x, y, z)	Laterality
Superior temporal sulcus	125	4.29	−54 −34 −2	Left
Inferior temporal sulcus		3.32	−54 −22 −20	Left
Superior temporal sulcus		3.19	−63 −25 −8	Left
Supramarginal gyrus	251	4.13	−45 −52 28	Left
Inferior parietal lobule		4.04	−48 −64 28	Left
Angular gyrus		3.54	−54 −58 28	Left

**Table 5.** Overview results aTL functional connectivity analysis

Anatomical location	Voxels	z value	MNI coordinates (x, y, z)	Laterality
Para-central lobule	149	3.99	12 −34 52	Right
Precuneus		3.13	12 −55 46	Right
Cingulate gyrus		3.11	12 −19 40	Right

## Discussion

We constantly predict others’ behavior and try to deduce their intentions during social interactions. Given the wide variability in traits and beliefs, it is difficult to model each individual’s mental state separately. One of the ways in which we simplify this process is by generalizing an individual’s behavior to their social group, and grounding our future predictions in these group preferences when predicting another individual’s behavior. While the brain regions associated with mentalizing an individual’s traits and desires have been identified and studied in great detail, the crucial question of how we learn to generalize these preferences, and then utilize this information when predicting individual information has remained elusive.

Our study employed a dynamic design in which we probed brain activity when predicting individual behavior based on learned group knowledge. This group knowledge was learned over the course of the experiment. Participants learned both a new stimulus set and rehearsed a previously learned stimulus set over the course of the functional MRI session. This type of design is generally better at dissociating the contributions of different parts of brain networks than pure comparison between static conditions. It has been successfully used in studies investigating learning of stimulus-response and reward associations (Toni *et al.*, 2001; Noonan *et al.*, 2011). In motor learning studies, it has been shown that retrieving overlearned associations relies on different brain networks that are active during learning itself (Toni *et al.*, 2002; Grol *et al.*, 2006). Even though performance at the end of the first day had gotten significantly better over trial blocks, we did not find a main effect of OLD *vs* NEW. Given the high number of trials used in the contrast (216 trials in each condition), it is unlikely that the results suffer from lack of power issue. It is more likely that participants were relatively fast in learning contingencies in the NEW condition. This is shown by the behavioral results of accuracy, where the main effect of CONDITION showed a very small effect size and no difference between NEW and OLD trials. However, we did find different dynamics of activation in different parts of the cortex.

The regions showing differential activation during the application of group knowledge generally broadly belonged to what has been termed the ‘social brain’, including the posterior part of the STS, TPJ and parts of medial frontal cortex (Saxe, 2006; Rushworth *et al.*, 2013; Schurz *et al.*, 2014; Molenberghs *et al.*, 2016). In general, these areas tended to show greater activation when applying group knowledge at the later stages of learning. Upon close inspection though, different parts of this larger network show subtle differences in the time course. Both in the posterior temporal and the medial frontal cortex, there is evidence of a gradient in learning dynamics, with different regions preferentially identified in the interaction contrast and in the overall time effect contrast.

Over the course of the scanning session, there was an increase in activation in both the OLD and NEW stimuli in the posterior cingulate and ventromedial frontal cortex. Both these activations were quite extensive and overlapped with the components of the default mode network (Raichle *et al.*, 2001) that is thought to often show activation in social cognition paradigms (Mars *et al.*, 2012a). In contrast, a more dorsal area of the medial frontal cortex overlapping with area 9 showed most activation only when applying group knowledge at the late stage of learning. A dorsal–ventral gradient of activation in the medial frontal cortex has been noted before. Although some studies ascribe a specific ‘social’ function to one or another of these areas, other studies argue for a difference based on the use rather than the type of information (Nicolle *et al.*, 2012). Such a dissociation could explain the different dynamics of learning the same type of information observed in the present study. Learning of knowledge of social groups based on observing individuals is consistent with some of the observations made of medial frontal regions in interactive settings. For instance, Stolk *et al.* (2015) used lesion data to demonstrate an essential role for ventromedial prefrontal cortex (vmPFC) in adjusting communication to the characteristics of the receiver. Dorsomedial PFC, on the other hand, has been shown to be involved in distinctions between self and other, maintaining representations of both (Wittmann *et al.*, 2016). One hypothesis arising from the current study is that this role could extend to maintaining or implementing dissociations between groups of different individuals in the same way as between self and others.

Areas along the STS, ranging from the anterior temporal cortex to the posterior STS and the so-called TPJ, are often identified in social tasks. TPJ especially has been associated with theory of mind (ToM) or mentalizing, i.e. the ability to represent the belief state of others (Saxe *et al.*, 2004b). These different parts of the temporal cortex are now known to differ both in their structural and functional connectivity (Mars *et al.*, 2012b; Xu *et al.*, 2016) and their functional profile (Schurz *et al.*, 2017). Consistent with this notion, they show slightly different dynamics here. The most posterior part of STS extending into TPJ was identified in the general late *vs* early contrast, while most anterior temporal areas were identified by the interaction contrast and most active when applying group knowledge during the last stage of OLD learning. TPJ is often thought to code more explicit, effortful social information, whereas the anteriorly located posterior STS might represent more automatic aspects of social information. Schurz and colleagues (2014) performed a meta-analysis of neuroimaging studies examining ToM tasks and observed that while the posterior TPJ was involved in most ToM tasks, anterior TPJ was activated in relatively simpler tasks like inferring the mental state of an individual based on a picture of their eyes. So, it is conceivable that as the stimuli become more overlearned they are processed more automatically and hence are preferentially coded here.

We also observed an increase in functional coupling between the temporal and medial cortex when applying group knowledge over the course of learning, over and above the changes in activation. In general, the regions increasing their coupling, vmPFC and TPJ on the one hand and aTL and posterior medial cortex on the other hand, are all regions that tend to belong to the same functional networks. For instance, in resting state fMRI one often identifies the default mode network discussed above that often is thought to include parts of TPJ (Schilbach *et al.*, 2008; Mars *et al.*, 2012a). Although aTL is not always included in this network, its functional connectivity profile with medial areas is similar to that of TPJ (Mars *et al.*, 2013), and there is evidence that the aTL plays a significant role in the processing of the ToM content (Ross and Olson, 2010).

While being part of the same mentalizing network, the mPFC and the TPJ are said to fulfill different roles. The mPFC is argued to be especially involved in processes related to the inferences of others’ traits and preferences that are stable over longer periods of time (Amodio and Frith, 2006; Kang *et al.*, 2013; Koster-Hale and Saxe, 2013), while the TPJ is implicated in processing the intentions of others and whether their behavior is consistent with these inferred intentions (Koster-Hale and Saxe, 2013; Koster-Hale *et al.*, 2013). Therefore, it is unlikely that the mPFC represents group knowledge itself, but more likely to make use of this knowledge when forming prediction of others’ preferences. Increased functional connectivity between mPFC and TPJ shows that forming predictions of others’ behavior based on group knowledge is, in part, facilitated by the mPFC–TPJ tandem. Although functional connectivity does not take causality into account, these results are in line with the MEG study that showed an increase in top-down influence of the mPFC on the TPJ when observing action kinematics and associated outcomes (van Pelt *et al.*, 2016). Here we suggest that the mPFC may use the stable trait of object-preferences given by group knowledge to form predictions of the preferences of an individual, based on which, the TPJ then generates predictions on the actual behavior of an individual.

One can raise the question whether our manipulation truly measures social predictions. Should we really care about other’s preferences in terms of clothing? While it is true that threat (‘kill my group’) or emotion (‘hate my group’) could likely result in bigger effect sizes, our aim was to answer questions independent of one’s own membership in the groups, thus avoiding in-group out-group confounds. More importantly, we wanted to first study how we acquire preferences at group-level and how this group-level knowledge is used to infer individual preferences in a way that reflects how we acquire this knowledge in daily lives. So, as a first step, we wanted to characterize the network of brain regions involved in this process. As a next step, it would certainly be interesting to study the effect of emotion on this process in a future study.

In this functional imaging study on learning and applying preferences of social groups to individuals, we established that the core regions identified in many aspects of social interactions with one individual extend their role to this generalizing function. This is not a trivial conclusion. Given that humans engage in larger social groups (Dunbar, 1992), it is likely that the skill to socially categorize people and learn their preferences at a group level, rather than at the individual level, is essential for us. Many of the regions identified here are present in some form in other primates (Rushworth *et al.*, 2012; Chang *et al.*, 2013). Our approach identifies some of the first differences in activation profiles of the various areas associated with learning social cognition at the level of groups, but the details remain to be teased apart. We present a number of hypotheses arising from these data that we hope can inspire future studies.

## Supplementary Material

nsaa136_Supp
