# Corrigendum to: Neural mechanisms of predicting individual preferences based on group membership

**DOI:** 10.1093/scan/nsab030

**Published:** 2021-03-12

**Authors:** Suhas Vijayakumar, Egbert Hartstra, Rogier B Mars, Harold Bekkering

**Affiliations:** Donders Institute for Brain, Cognition and Behaviour, Radboud University Nijmegen, HR, Nijmegen, The Netherlands; Donders Institute for Brain, Cognition and Behaviour, Radboud University Nijmegen, HR, Nijmegen, The Netherlands; Donders Institute for Brain, Cognition and Behaviour, Radboud University Nijmegen, HR, Nijmegen, The Netherlands; Wellcome Centre for Integrative Neuroimaging, Centre for Functional MRI of the Brain (FMRIB), Nuffield Department of Clinical Neurosciences, John Radcliffe Hospital, University of Oxford, Oxford, United Kingdom; Donders Institute for Brain, Cognition and Behaviour, Radboud University Nijmegen, HR, Nijmegen, The Netherlands

In the originally published version of this manuscript, significant errors were noted and listed in this corrigendum.

Figure 1 has been updated as follows:

Previous version


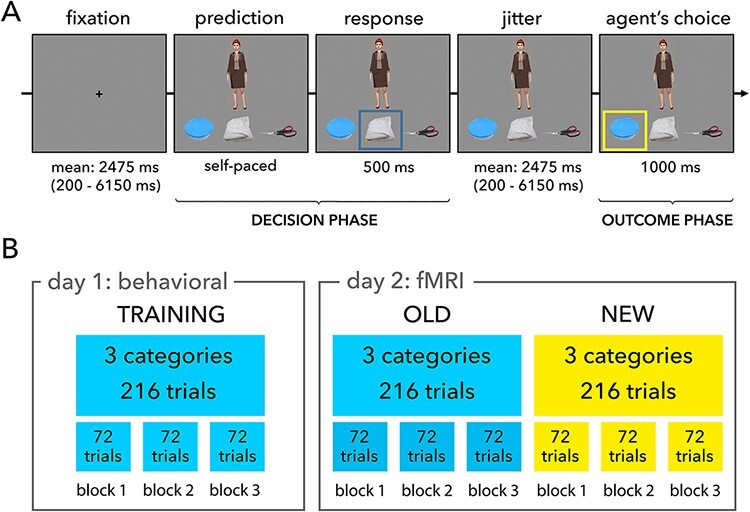


Corrected version 
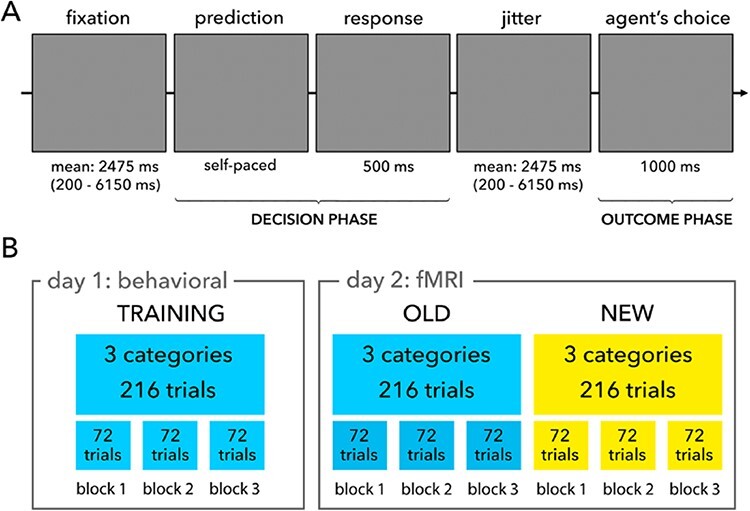


The legend of ‘Figure 1A’ inadvertently omitted the following sentence: ‘Note: The agent depicted in this figure closely resembles one of the agents used in the experiment and is not from the actual stimulus set.’

In the Methods section, the following corrections were made:

Under the ‘Stimulus material’ heading, the following text should read:

‘with 12 agents in each social-group. The virtual agents were designed using SIMS 4 (Electronic Arts Inc., 2014) and their group consistency was verified by means of a separate pilot experiment (for more details, see supplementary material, section “stimulus validation”). All virtual agents were white females and were portrayed in the same posture, to avoid possible confounds arising from such differences. An equal number of object categories were created with 12 objects in each category by means of photos of 108 objects (see supplementary material, section “stimulus material”. Supplementary Figure S2 contains all of the object images used in the experiment), so that each social group could’ instead of:

‘with 12 agents in each social-group (see supplementary material, section “stimulus material” for an overview of all of the stimulus material used in the study). The virtual agents were designed using SIMS 4 (Electronic Arts Inc., 2014) and their group consistency was verified by means of a separate pilot experiment (for more details see supplementary material, section “stimulus validation”). All virtual agents were white females, and were portrayed in the same posture, to avoid possible confounds arising from such differences. An equal number of object categories were created with 12 objects in each category by means of photos of 108 objects, so that each social group could’.

Under the ‘Trial structure’ heading, the following text was inadvertently omitted from the first paragraph: ‘(note that the agent shown in Figure 1A closely resembles one of the agents used in the experiment)’.

Under the ‘fMRI analyses’ heading, the last sentence in the last paragraph should read: ‘The resulting activation maps were tested for significance at a voxel level threshold of *P* < 0.001 (uncorrected) while correcting for multiple comparisons using a family-wise error (FWE) cluster-corrected probability of *P* < 0.05.’ instead of ‘The resulting activation maps were tested for significance at a voxel level threshold of *P* < 0.001 (uncorrected) while correcting for multiple comparisons using an family-wise error (FWE) cluster-corrected probability of *P* < 0.05.’

In the Results section, the following corrections were made:

The figure reference to ‘Supplementary Figure S4’ in the first paragraph under the ‘Behavioral results’ heading, should read: ‘Supplementary Figure S3’.

Under the ‘fMRI results’ heading, the following text was inadvertently omitted from the last paragraph: ‘(under section “parametric modulation results”)’.

In the Supplementary data, the following corrections were made:

Under the ‘Stimulus material’ heading, corrections were made to the introductory text and the legend of Supplementary Figures S1 and S2. Supplementary Figure S3 was removed. As a result, under the ‘Behavioral results’ heading, reference to ‘Supplementary Figure S4A’ under the ‘Reaction time’ sub-heading should read: ‘Supplementary Figure S3A’. Under the ‘Accuracy’ sub-heading, reference to ‘Supplementary Figure S4B’ in the first paragraph should read: ‘Supplementary Figure S3B’. In addition, the legend title ‘Supplementary Figure S4’ should read: ‘Supplementary Figure S3’. Moreover, the legend titles and figure in-text reference for ‘Supplementary Figure S5’ and ‘Supplementary Figure S6’ were also changed.

Under the ‘Materials’ sub-heading for the ‘Methods’ heading, the following text should read: ‘“artistic black”, and “punk”. All the aspects related to clothing style’ instead of ‘“artistic black”, and “punk” (see Supplementary Figure S1A for a representative agent from each of these categories, or Supplementary Figure S2 for an overview of all agents categories, and Supplementary Figure S3 for all object categories). All the aspects related to clothing style’.

These errors have now been corrected.

