# Supplementary material for: Neural mechanisms of predicting individual preferences based on group membership
Source: Soc Cogn Affect Neurosci. 2020 Oct 7;16(9):1006–17. doi: 10.1093/scan/nsaa136 (PMC8421698; doi:10.1093/scan/nsaa136)
Supplement: nsaa136_Supp [file nsaa136_supp.zip › CORRECTED_SCAN_SUPP_TEXT_learning_group_membership_ONLY_FOR_YOUR_REFERENCE.docx]

Supplementary Material

Neural mechanisms of predicting individual preferences based on group membership

Suhas Vijayakumar*^1^, Egbert Hartstra*^1^, Rogier B. Mars^1,2^, Harold Bekkering^1^

**Running title**: applying learned group knowledge

**Correspondence**: Suhas Vijayakumar [vijayakumar.suhas@gmail.com](mailto:vijayakumar.suhas@gmail.com)

Postbus 9104, 6500 HE Nijmegen, The Netherlands. Phone: +31-24-3612689

*These authors contributed equally to this work

^1^ Donders Institute for Brain, Cognition and Behaviour, Radboud University Nijmegen, 6525HR Nijmegen, The Netherlands.

^2^ Wellcome Centre for Integrative Neuroimaging, Centre for Functional MRI of the Brain (FMRIB), Nuffield Department of Clinical Neurosciences, John Radcliffe Hospital, University of Oxford, Headington, Oxford OX3 9DU, Oxford, United Kingdom.

# Stimulus material

A representative stimulus from each of the nine object categories and the full set of object stimulus material is provided below (Fig. S1 and Fig. S2).


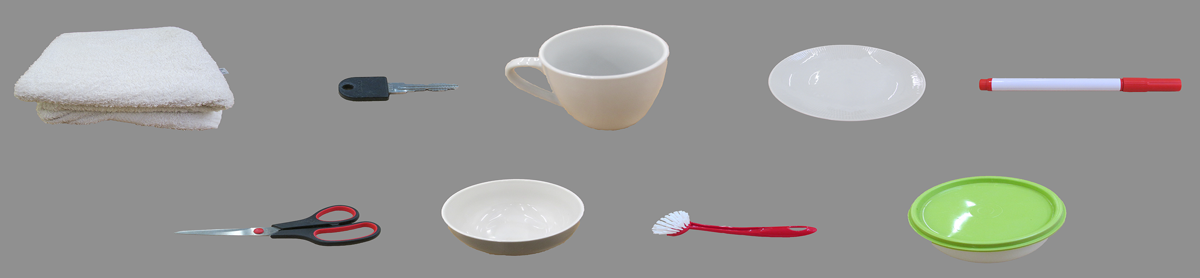


**Figure S1.** A representative sample from each of the object categories. In order from left to right: towels, keys, cups, plates, marker pens, scissors, bowls, cleaning brushes, and tupperware.


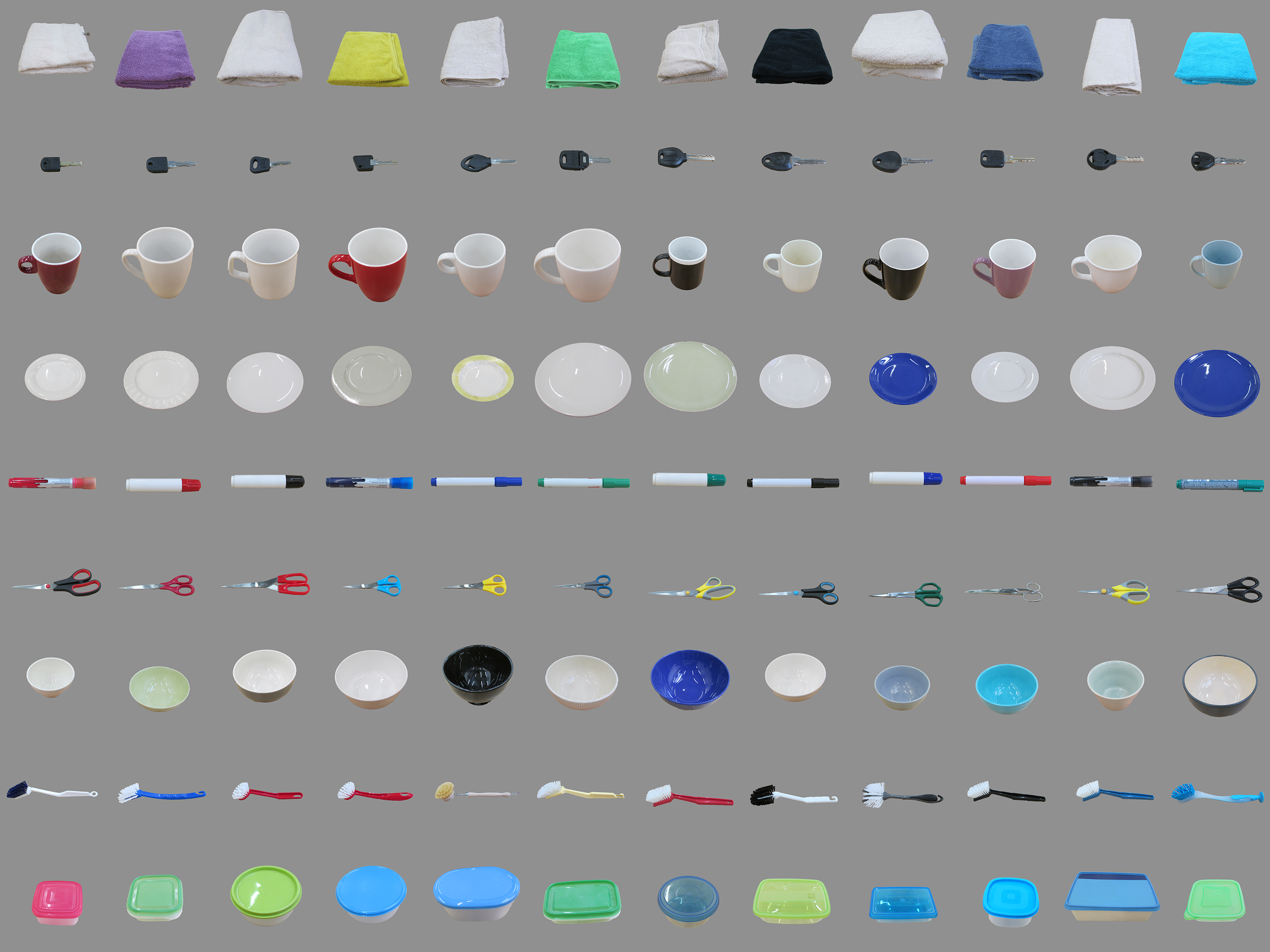


**Figure S2.** All objects in object categories. Each row represents an object category.
Top to bottom: towels, keys, cups, plates, marker pens, scissors, bowls, cleaning brush, and tupperware.

# Trial distribution

A mock distribution of 72 trials of an agent category is shown in Table S1. Number of times an agent chose an object from the most preferred category is marked as “1” in green. Number of times an agent chose an object from the less preferred category is marked “2” in orange, and the number of times an agent chose an object from the least preferred category is marked as “3” in red.

**Table S1.** An example of 72 trials of an agent category, and their chosen object category preferences.

| Agents in each agent category | | | | | | | | | | | |
| --- | --- | --- | --- | --- | --- | --- | --- | --- | --- | --- | --- |
| 1 | 2 | 3 | 4 | 5 | 6 | 7 | 8 | 9 | 10 | 11 | 12 |
| 1 | 1 | 1 | 1 | 1 | 1 | 1 | 1 | 1 | 1 | 1 | 1 |
| 1 | 1 | 1 | 1 | 1 | 1 | 1 | 1 | 1 | 1 | 1 | 1 |
| 1 | 1 | 1 | 1 | 1 | 1 | 1 | 1 | 1 | 1 | 1 | 1 |
| 1 | 1 | 1 | 1 | 1 | 1 | 1 | 1 | 1 | 2 | 2 | 2 |
| 2 | 2 | 2 | 2 | 2 | 2 | 2 | 2 | 2 | 2 | 2 | 2 |
| 2 | 2 | 2 | 3 | 3 | 3 | 3 | 3 | 3 | 3 | 3 | 3 |
|  | | | | | | | | | | | |
| preference | | | 1 | preference | | | 2 | preference | | | 3 |

#

# Stimulus validation (agent categories)

We investigated if our individual agents could be successfully grouped into intended categories by conducting an online study. We decided to not use gender or racial categories as a way to group these individuals because these categories might be learned faster than other social categories and may lead to biases. Also, participants could already have associations for these types of groups, which would be a confound in studying how we learn new group preferences. Therefore, we chose to use different types of clothing style as a way to categorize. We decided on a list of social groups based on clothing style and created agents for each of these groups. Participants were given a list with pictures of the individual agents and were asked to put them into different social groups. We expected that the participants would categorize these agents based on the intended clothing style, while ignoring other physical appearance characteristics such as height or eye color.

## Methods

### Participants

In total, 31 participants (30 females; 18-24y, M = 19.71y, SD = 1.64y) completed the survey. Another four started the survey but dropped out. Every participant was a student at Radboud University Nijmegen and was rewarded with course credits upon completing the survey. The study was approved by the Ethical Commission of Donders Centre for Cognition. Participants signed an informed consent form before the study.

### Design

A questionnaire was administered in which participants had to categorize pictures of agents. They were presented with a list of 48 or 60 pictures, and were asked to group these pictures into four or five categories respectively. The number of groups they had to make was fixed in advance, but crucially, no information was given on what types of groups they could make. As such, it could investigate whether participants recognized our social groups along with whether they could ignore the characteristics that were irrelevant to our social groups.

### Materials

The agents had been created using the ‘Create-a-Sim’ toolbox in the video game The Sims 4 (EA Maxis & The Sims Studio, 2014). In total, nine different social categories were used: ‘short pants’, ‘long dresses’, ‘office-wear’, ‘sporty’, ‘tattooed’, ‘casual’, ‘summer dress’, ‘artistic black’, and ‘punk’. All the aspects related to clothing style – clothes, shoes, make-up, hair, and accessories – were fit to the agent’s social groups. Importantly, agents of different categories could not be distinguished on the basis of one single feature (e.g. shoes); the complete picture was needed to give a reliable judgement. All the created agents were female Caucasian young adults. Other physical characteristics, such as eye colour or body stance, did not hold any relation to the different social groups. The questionnaire was developed and administered in Qualtrics (2014) using a university-wide online testing platform.

###

### Procedure

After seeing a welcome and instruction screen, and after filling out their background information (gender and age), participants were asked to categorize pictures of individuals into different social groups. This part of the questionnaire consisted of two blocks. In one block, 48 pictures of agents had to be categorized into four different groups. In the other block, 60 pictures of agents had to be categorized into five different groups. In both blocks, participants were required to use all boxes. They were allowed to group in an unbalanced manner; no limitations had been set on how many pictures each group should contain. No further information was given on what types of groups were expected. The order of the two blocks was counterbalanced across participants.

On the left side of the screen, participants were presented with the list of 48 or 60 pictures, while on the right side, they were presented with the respectively four or five empty boxes. Participants could drag the pictures to the boxes one by one. The order of pictures in the lists was randomised. To make sure not all participants had the same lists of stimuli, five different versions of the questionnaire were made. In each version, the social categories were randomly assigned. The different versions were put online one at the time: whenever a version had at least six complete responses, it was closed, and the next version was put online. Each of the version had six full responses - except the second version, which had seven full responses.

### Data coding

As all participants received a different order of pictures and since multiple versions of the questionnaire were available, participants had different orders of the groups. Recoding was therefore required. For all nine groups that participants had made, it was decided which of the social groups was most prominent. For example, if a participant had made a group with seven pictures of the ‘long dresses’ group and two pictures of the ‘summer dresses’ group, this category was denoted as the long dresses group. And if a participant had made a group with four pictures of the ‘office-wear’ group and four of the ‘short pants’ group, but had made another group with five pictures of the ‘office-wear’ group, the groups were coded as ‘short pants’ and ‘office-wear’ respectively. Next, pictures were scored for each participant: if a picture was grouped in its original category, it was rated as correct (1), while if it was grouped in one of the other categories, it was scored as incorrect (0). Next, a total score was calculated for each of the categories; on each category, participants could get anything between zero and twelve correct items.

## Results

To see if there was any difference in performance between the categories, a Repeated Measures (RM) ANOVA was performed on the total score of each category (1-9). This analysis showed there were differences between the total scores of the nine categories, F(8, 23) = 4.23, p = .003, η^2^ = .60. However, after correcting for multiple testing (Bonferroni, α = .05), we found there was no category in which participants performed differently compared to the other categories (p > .05).

Reliability analysis were performed on these 0-1 scores to see if the social groups had good internal consistency, and to see if all items fit well in the groups – reflecting to what extent participants recognized the different social groups. Results are summarised in Table S2. All categories showed high internal consistency and had high proportions correct. Most of the items had either a detrimental effect on Cronbach’s α if deleted, and if deleting the item increased Cronbach’s α, the effect was usually very low (increases lower than .01). A few exceptions could be seen. In the tattoos-category, one of the items had a negative effect on the internal consistency, and had an item-rest correlation of .00. Further inspection showed this was caused by the fact that the item was always grouped correctly (proportion correct = 1.00). In the casual-category, one of the items had a Cronbach’s α if deleted value of .925, while the original α was .897. Item-rest correlation of this item was -.023.

**Table S2.** Summary of internal consistency values for each social category.

| **Social**  **category** | **Cronbach’s**  **α** | **Cronbach’s α if deleted**  **[min-max]** | **Proportions**  **correct [mean]** | **Proportions**  **correct [min-max]** |
| --- | --- | --- | --- | --- |
| Office-wear | 0.879 | .854 – .886 | 0.67 | .61 – .77 |
| Artistic black | 0.957 | .949 – .958 | 0.7 | .58 – .81 |
| Sporty | 0.904 | .888 – .909 | 0.85 | .74 – .90 |
| Casual | 0.897 | .877 – .925 | 0.75 | .55 – .87 |
| Summer dresses | 0.913 | .900 – .910 | 0.78 | .71 – .84 |
| Long dresses | 0.931 | .917 – .940 | 0.87 | .71 – .97 |
| Short pants | 0.928 | .914 – .930 | 0.64 | .58 – .77 |
| Tattooed | 0.936 | .926 – .943 | 0.9 | .74 – 1.0 |
| Punk | 0.918 | .901 – .919 | 0.77 | .68 – .94 |

In conclusion, participants were able to recognize the social categories that we had created: when comparing the groups they had made to the intended groups, a large proportion of items were correctly categorized. The social categories had high internal consistency, and no differences were found on the performance between the categories for each participant. Two items were found to be inconsistent with their groups. The first one, in the tattoo-category, was inconsistent because it was easier than most items – therefore, no changes were necessary. The other item, in the casual category, was adjusted to better fit with the rest of the social group.

# Stimulus set: agent category and their respective object category preferences for each participant

Agent categories and their respective object category preferences used in the experiment for each participant is provided in table S3, including agent-object pairings for participants who were excluded from the analyses.

**Table S3.** Agent categories and their respective object category preferences used in the experiment for each participant.

| **Participant** | **Condition** | **Agent category** | **Object category** | | |
| --- | --- | --- | --- | --- | --- |
|  |  |  | **Preference 1** | **Preference 2** | **Preference 3** |
| 1 | OLD | artistic black | cleaning brush | tupperware | towels |
|  |  | summer dress | towels | cleaning brush | tupperware |
|  |  | long dresses | tupperware | towels | cleaning brush |
|  | NEW | sporty | scissors | cups | bowls |
|  |  | punk | cups | bowls | scissors |
|  |  | short pants | bowls | scissors | cups |
| 2 | OLD | short pants | keys | cups | tupperware |
|  |  | casual | cups | tupperware | keys |
|  |  | sporty | tupperware | keys | cups |
|  | NEW | tattooed | marker pens | scissors | towels |
|  |  | long dresses | scissors | towels | marker pens |
|  |  | office-wear | towels | marker pens | scissors |
| 3 | OLD | office-wear | tupperware | towels | cleaning brush |
|  |  | casual | towels | cleaning brush | tupperware |
|  |  | artistic black | cleaning brush | tupperware | towels |
|  | NEW | tattooed | keys | scissors | bowls |
|  |  | long dresses | bowls | keys | scissors |
|  |  | short pants | scissors | bowls | keys |
| 4 | OLD | office-wear | scissors | tupperware | towels |
|  |  | tattooed | towels | scissors | tupperware |
|  |  | punk | tupperware | towels | scissors |
|  | NEW | sporty | marker pens | bowls | cups |
|  |  | summer dress | cups | marker pens | bowls |
|  |  | artistic black | bowls | cups | marker pens |
| 5 | OLD | sporty | cups | bowls | keys |
|  |  | office-wear | bowls | keys | cups |
|  |  | summer dress | keys | cups | bowls |
|  | NEW | long dresses | cleaning brush | marker pens | tupperware |
|  |  | short pants | tupperware | cleaning brush | marker pens |
|  |  | artistic black | marker pens | tupperware | cleaning brush |
| 6 | OLD | artistic black | marker pens | cups | scissors |
|  |  | punk | cups | scissors | marker pens |
|  |  | long dresses | scissors | marker pens | cups |
|  | NEW | casual | towels | plates | bowls |
|  |  | short pants | bowls | towels | plates |
|  |  | tattooed | plates | bowls | towels |
| 7 | OLD | punk | cups | towels | scissors |
|  |  | short pants | scissors | cups | towels |
|  |  | artistic black | towels | scissors | cups |
|  | NEW | office-wear | bowls | keys | tupperware |
|  |  | sporty | keys | tupperware | bowls |
|  |  | summer dress | tupperware | bowls | keys |
| 8 | OLD | summer dress | cups | marker pens | scissors |
|  |  | sporty | scissors | cups | marker pens |
|  |  | office-wear | marker pens | scissors | cups |
|  | NEW | punk | bowls | towels | keys |
|  |  | artistic black | keys | bowls | towels |
|  |  | casual | towels | keys | bowls |
| 9 | OLD | summer dress | marker pens | towels | keys |
|  |  | punk | keys | marker pens | towels |
|  |  | tattooed | towels | keys | marker pens |
|  | NEW | sporty | bowls | cups | cleaning brush |
|  |  | artistic black | cleaning brush | bowls | cups |
|  |  | short pants | cups | cleaning brush | bowls |
| 10 | OLD | long dresses | towels | cleaning brush | scissors |
|  |  | tattooed | cleaning brush | scissors | towels |
|  |  | office-wear | scissors | towels | cleaning brush |
|  | NEW | short pants | cups | bowls | keys |
|  |  | sporty | bowls | keys | cups |
|  |  | casual | keys | cups | bowls |
| 11 | OLD | long dresses | marker pens | towels | keys |
|  |  | casual | keys | marker pens | towels |
|  |  | summer dress | towels | keys | marker pens |
|  | NEW | artistic black | plates | cleaning brush | scissors |
|  |  | sporty | scissors | plates | cleaning brush |
|  |  | punk | cleaning brush | scissors | plates |
| 12 | OLD | short pants | marker pens | keys | cleaning brush |
|  |  | long dresses | cleaning brush | marker pens | keys |
|  |  | sporty | keys | cleaning brush | marker pens |
|  | NEW | tattooed | towels | bowls | scissors |
|  |  | casual | bowls | scissors | towels |
|  |  | summer dress | scissors | towels | bowls |
| 13 | OLD | office-wear | cups | cleaning brush | keys |
|  |  | tattooed | cleaning brush | keys | cups |
|  |  | summer dress | keys | cups | cleaning brush |
|  | NEW | long dresses | scissors | plates | marker pens |
|  |  | sporty | plates | marker pens | scissors |
|  |  | punk | marker pens | scissors | plates |
| 14 | OLD | artistic black | tupperware | plates | marker pens |
|  |  | sporty | plates | marker pens | tupperware |
|  |  | office-wear | marker pens | tupperware | plates |
|  | NEW | long dresses | bowls | cleaning brush | towels |
|  |  | tattooed | towels | bowls | cleaning brush |
|  |  | punk | cleaning brush | towels | bowls |
| 15 | OLD | sporty | bowls | cups | keys |
|  |  | short pants | keys | bowls | cups |
|  |  | casual | cups | keys | bowls |
|  | NEW | office-wear | plates | marker pens | scissors |
|  |  | long dresses | marker pens | scissors | plates |
|  |  | tattooed | scissors | plates | marker pens |
| 16 | OLD | casual | bowls | cups | tupperware |
|  |  | summer dress | tupperware | bowls | cups |
|  |  | artistic black | cups | tupperware | bowls |
|  | NEW | tattooed | towels | plates | keys |
|  |  | short pants | keys | towels | plates |
|  |  | punk | plates | keys | towels |
| 17 | OLD | tattooed | keys | tupperware | scissors |
|  |  | casual | tupperware | scissors | keys |
|  |  | summer dress | scissors | keys | tupperware |
|  | NEW | sporty | bowls | marker pens | plates |
|  |  | short pants | plates | bowls | marker pens |
|  |  | office-wear | marker pens | plates | bowls |
| 18 | OLD | office-wear | marker pens | bowls | tupperware |
|  |  | short pants | tupperware | marker pens | bowls |
|  |  | artistic black | bowls | tupperware | marker pens |
|  | NEW | casual | scissors | cups | towels |
|  |  | punk | towels | scissors | cups |
|  |  | summer dress | cups | towels | scissors |
| 19 | OLD | summer dress | keys | towels | scissors |
|  |  | tattooed | scissors | keys | towels |
|  |  | long dresses | towels | scissors | keys |
|  | NEW | casual | cleaning brush | tupperware | marker pens |
|  |  | short pants | tupperware | marker pens | cleaning brush |
|  |  | sporty | marker pens | cleaning brush | tupperware |
| 20 | OLD | summer dress | towels | keys | marker pens |
|  |  | casual | keys | marker pens | towels |
|  |  | artistic black | marker pens | towels | keys |
|  | NEW | short pants | bowls | scissors | cups |
|  |  | tattooed | cups | bowls | scissors |
|  |  | long dresses | scissors | cups | bowls |
| 21 | OLD | punk | cups | marker pens | tupperware |
|  |  | sporty | marker pens | tupperware | cups |
|  |  | short pants | tupperware | cups | marker pens |
|  | NEW | artistic black | towels | keys | plates |
|  |  | office-wear | keys | plates | towels |
|  |  | casual | plates | towels | keys |
| 22 | OLD | summer dress | cups | cleaning brush | plates |
|  |  | sporty | cleaning brush | plates | cups |
|  |  | short pants | plates | cups | cleaning brush |
|  | NEW | casual | keys | bowls | tupperware |
|  |  | punk | tupperware | keys | bowls |
|  |  | artistic black | bowls | tupperware | keys |
| 23 | OLD | punk | towels | cups | cleaning brush |
|  |  | long dresses | cups | cleaning brush | towels |
|  |  | artistic black | cleaning brush | towels | cups |
|  | NEW | short pants | marker pens | scissors | keys |
|  |  | summer dress | scissors | keys | marker pens |
|  |  | casual | keys | marker pens | scissors |
| 24 | OLD | office-wear | keys | towels | scissors |
|  |  | punk | scissors | keys | towels |
|  |  | long dresses | towels | scissors | keys |
|  | NEW | artistic black | cups | cleaning brush | plates |
|  |  | sporty | cleaning brush | plates | cups |
|  |  | short pants | plates | cups | cleaning brush |
| 25 | OLD | casual | cleaning brush | keys | bowls |
|  |  | long dresses | keys | bowls | cleaning brush |
|  |  | summer dress | bowls | cleaning brush | keys |
|  | NEW | artistic black | scissors | marker pens | cups |
|  |  | sporty | cups | scissors | marker pens |
|  |  | office-wear | marker pens | cups | scissors |
| 26 | OLD | tattooed | tupperware | plates | scissors |
|  |  | office-wear | scissors | tupperware | plates |
|  |  | sporty | plates | scissors | tupperware |
|  | NEW | short pants | marker pens | cups | towels |
|  |  | casual | cups | towels | marker pens |
|  |  | long dresses | towels | marker pens | cups |
| 27 | OLD | punk | towels | tupperware | cups |
|  |  | artistic black | cups | towels | tupperware |
|  |  | long dresses | tupperware | cups | towels |
|  | NEW | casual | plates | marker pens | cleaning brush |
|  |  | short pants | cleaning brush | plates | marker pens |
|  |  | tattooed | marker pens | cleaning brush | plates |

#

# Methods

## Participant exclusion criteria

During each trial, participants either correctly identified the object of first preference, or not. Using the Binomial test we could identify whether the probability of observing a certain number of successes for each participant was significantly different from chance. So, after excluding participants who could not complete the experiment, we performed a one-sided Binomial test on 24 participants using a Matlab function called “myBinomTest” (<https://www.mathworks.com/matlabcentral/fileexchange/24813-mybinomtest-s-n-p-sided>).

Responses for 216 trials of NEW condition were used to test successful learning. Table S4 summarizes these results.

**Table S4.** Summary of one-sided Binomial test (*n*=24)

| **Participant** | **p-value** | **different from chance?** |
| --- | --- | --- |
| 1 | **0.389** | **not significant** |
| 2 | < 0.05 | significant |
| 3 | < 0.05 | significant |
| 4 | < 0.05 | significant |
| 5 | < 0.05 | significant |
| 6 | < 0.05 | significant |
| 7 | < 0.05 | significant |
| 8 | < 0.05 | significant |
| 9 | < 0.05 | significant |
| 10 | < 0.05 | significant |
| 11 | < 0.05 | significant |
| 12 | < 0.05 | significant |
| 13 | < 0.05 | significant |
| 14 | < 0.05 | significant |
| 15 | < 0.05 | significant |
| 16 | < 0.05 | significant |
| 17 | < 0.05 | significant |
| 18 | < 0.05 | significant |
| 19 | < 0.05 | significant |
| 20 | **0.089** | **not significant** |
| 21 | < 0.05 | significant |
| 22 | < 0.05 | significant |
| 23 | < 0.05 | significant |
| 24 | < 0.05 | significant |

# Behavioral results

## Reaction time

On examining mean reaction time (RT) during fMRI session, we found a significant main effect of CONDITION (*F*(1, 21) = 8.535, *p* < 0.01, *η_p_²* = 0.289) as well as a significant main effect of BLOCK (*F*(2, 42) = 17.6, *p* < 0.001, *η_p_²* = 0.456) (Fig. S3 A). However, the interaction effect was not significant (*F*(2, 42) = 2.057, *p* = 0.140, *η_p_²* = 0.089). During the NEW trials, mean RT was not significantly different (*p* = 0.061) between BLOCK1 (*M* = 2.166, *SD* = 0.736) and BLOCK2 (*M* = 1.818, *SD* = 0.436). Whereas it significantly decreased (*p* < 0.05) from BLOCK2 to BLOCK3 (*M* = 1.681, *SD* = 0.345). Mean RT of BLOCK3 was also significantly different from that of BLOCK1 (*p* < 0.01).

During the OLD trials, mean RT of BLOCK1 (*M* = 1.824, *SD* = 0.450) was only significantly different (*p* < 0.05) from that of mean RT of BLOCK3 (*M* = 1.608, *SD* = 0.485). And RTs during BLOCK2 (*M* = 1.706, *SD* = 0.527) were neither significantly different (*p* = 0.329) from BLOCK1, nor from BLOCK3 (*p* = 0.238).

## Accuracy

It does not follow from a significant reduction in RTs, that the participants were able to deduce object-category preferences for given agent-categories. To determine whether they learned to respond with the most preferred object category for each agent category, repeated measures ANOVA was performed on average accuracy scores of participants. Accuracy scores were the percentage of correct trials in each block, with correct trials defined as the trials in which participants chose the object from first preference category. Results showed no significant effect of CONDITION (*F*(1,21) = 1.306, *p* = 0.266, *η_p_²* = 0.059), but did show a significant main effect of BLOCK (*F*(2,42) = 18.539, *p* < 0.001, *η_p_²* = 0.469) and a significant interaction effect of CONDITION × BLOCK (*F*(2,42) = 8.177, *p* < 0.01, *η_p_²* = 0.280) (Fig. S3 B). Upon performing Bonferroni corrected post-hoc tests on NEW trials, it was found that the accuracy was significantly different (*p* < 0.001) between BLOCK1 (*M* = 76.203, *SD* = 18.086) and BLOCK2 (*M* = 85.190, *SD* = 17.815), and also significantly different (*p* < 0.01) between BLOCK1 and BLOCK3 (*M* = 84.226, *SD* = 21.198). But there was no significant difference between BLOCK2 accuracy and BLOCK3 accuracy (SPSS Bonferroni adjusted *p* = 1).

During OLD trials, interestingly, there was no significant difference between accuracy scores of any of the three BLOCKs; no significant difference (SPSS Bonferroni adjusted *p* = 1) between BLOCK1 (*M* = 84.820, *SD* = 17.628) and BLOCK2 (*M* = 86.157, *SD* = 18.096), between BLOCK2 and BLOCK3 (*M* = 86.924, *SD* = 17.782) (SPSS Bonferroni adjusted *p* = 1) and between BLOCK1 and BLOCK3 (*p* = 0.380).


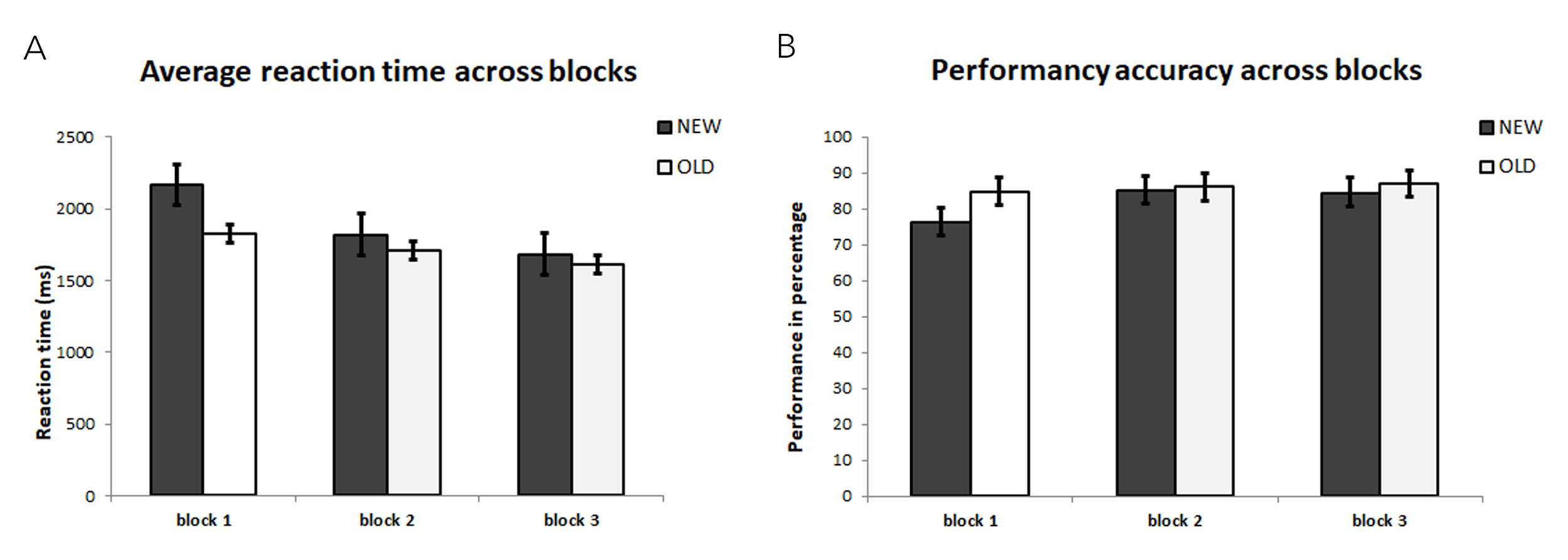


**Figure S3 A.** Average RTs of participants over time blocks. Error bars represent standard error of the mean (*n*=22). **B.** Performance accuracy of participants over blocks during fMRI session. Error bars represent standard error of the mean (*n*=22).

# Beta estimates of regions involved in overall time effect contrast

Beta estimates of regions involved in overall time effect contrast are provided below (Fig. S4). MNI coordinates of activation peaks are listed in table 2.


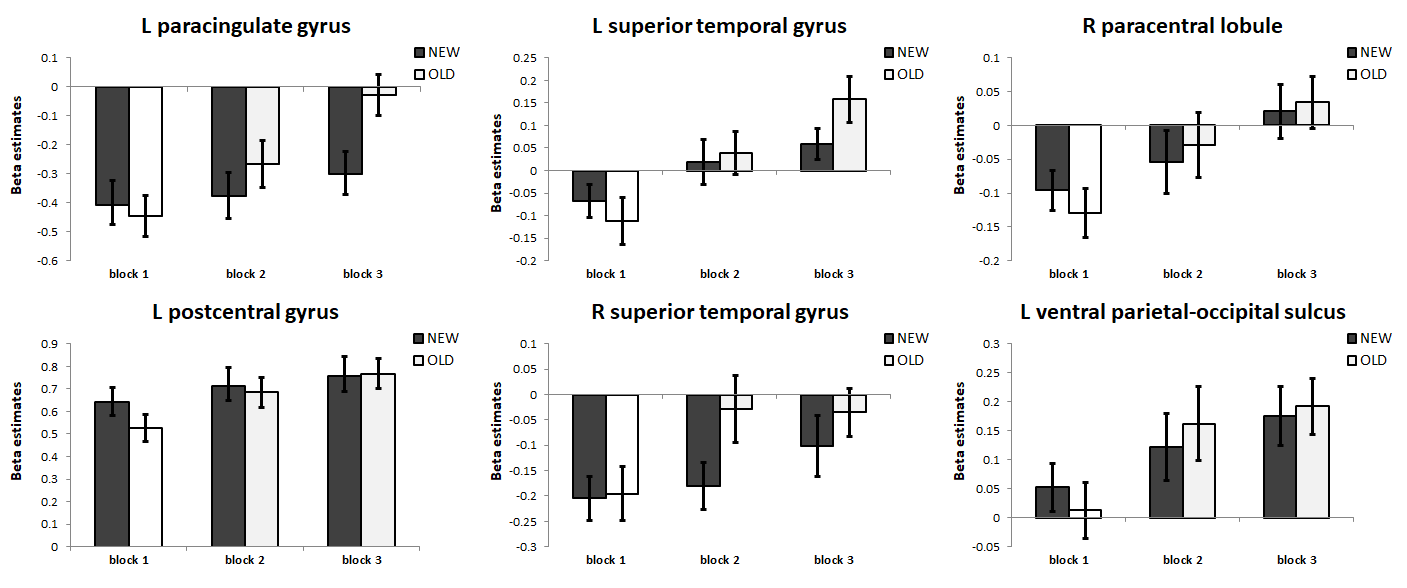


**Figure S4.** Beta estimates of regions involved in overall time effect contrast (BLOCK3>BLOCK1).

#

# Parametric modulation results

## Trial-by-trial efficiency indices

To represent how a participant’s performance varied over time, we estimated trial-by-trial efficiency indices throughout the experiment session. In order to do so and to ensure that the data satisfies the assumption of normality, we calculated efficiency indices per trial by using a moving average of seven trials (consisting of three trials before current trial, current trial, and three trials after) (see Stolk et al., [(2014)](https://paperpile.com/c/858tAS/DvfL/?noauthor=1) for a similar analysis).This resulted in trial-by-trial efficiency indices, in which higher scores reflected better performance on the task. These trial-by-trial efficiency indices were included as a parametric modulator for each block in the GLM used to investigate the decision phase (see methods section of the main text for information about other regressors). Contrast images were computed for the parametric modulation regressor for each participant. Thereafter, individual contrast images were submitted to a second level group analyses using t-test with cluster threshold of *p* < 0.001 (uncorrected) and cluster probability of threshold of *p* < 0.05, FEW corrected for multiple comparisons.

## fMRI results

Participants acquired information about agent-object preferences upon observing evidence multiple times throughout the experiment and hence, progressively learnt to make better predictions. We were able to associate a learning parameter value for each trial by calculating average over a moving 7-trial smoothing window of efficiency index scores. Results showed significant increase in activation in bilateral visual cortex spanning V1, V2, and V4, in area 7a of the left superior parietal lobule, in left anterior intraparietal sulcus, and in the left inferior parietal lobule (supplementary figure S5, supplementary table S5). Thus, trail-by-trial fluctuations in task performance are linked to increased involvement of visual areas and parietal areas, while the mPFC, TPJ and aTL show a general increase in involvement over the course of the experiment. This presents an interesting dissociation between the systems involved in overall learning of the task structure and those involved in the processing of the trial-by-trial information. As the task is probabilistic, the overall structure of learning is best picked up with a model of the general changes over time, while the implementation of the task is better picked up by a trial-by-trial model. The mPFC, along with TPJ, has been shown to be involved in inferring traits of other people over long-term (Koster-Hale and Saxe, 2013). During the “decision phase” of the experiment, participants use the mentalizing network to make predictions about an individual’s preference based on group membership after they have acquired this information. Results from the parametric modulation analysis, the trial-by-trial model, show that the visual and parietal areas process the immediate trial-specific information needed to perform the task throughout the experiment, even when they are yet to acquire group memberships. Further investigation of the “outcome phase” of our study will inform us of how this trial-by-trial information is used in learning preferences of group memberships.


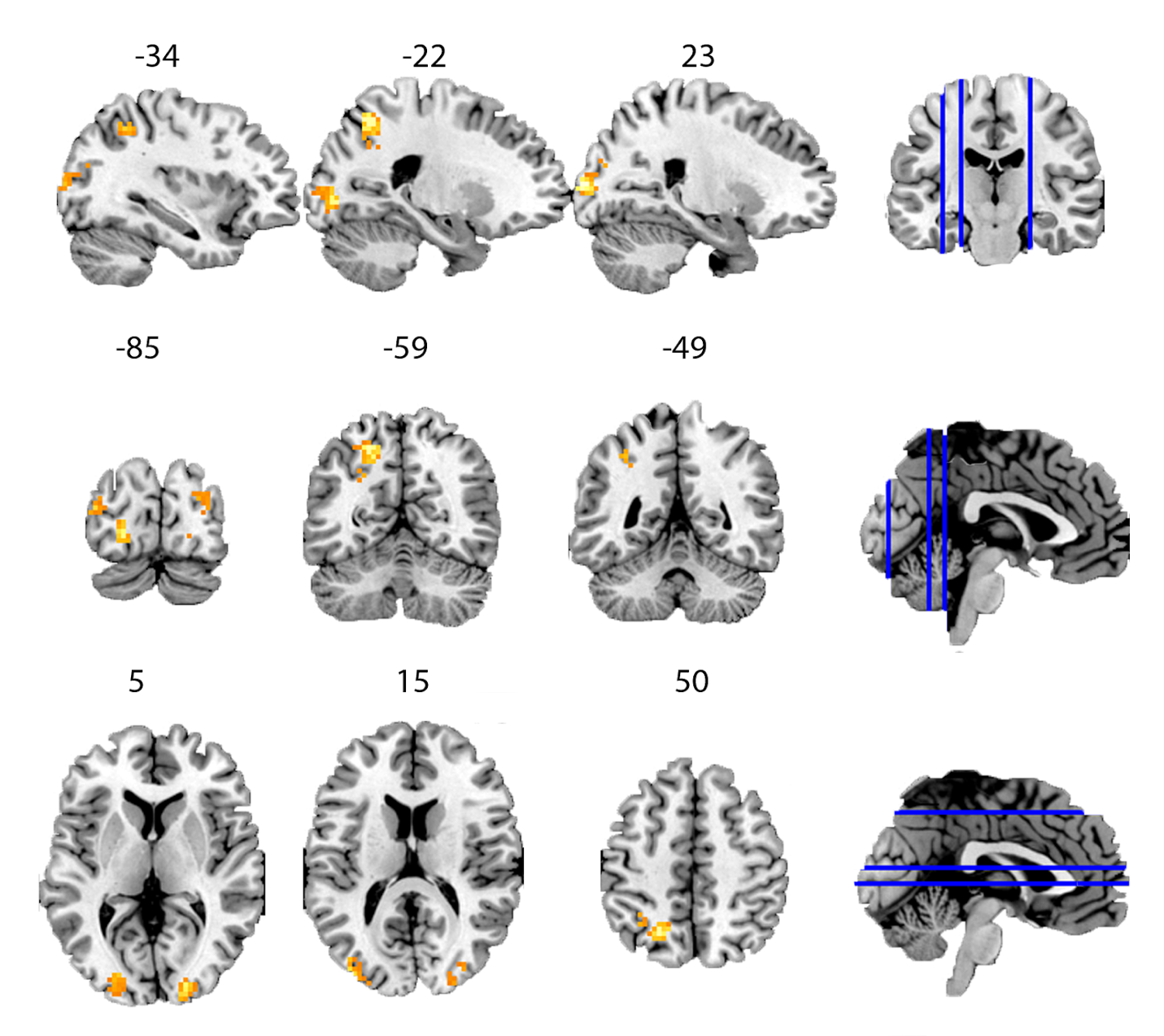


**Figure S5.** Whole-brain activation map of parametric modulation analysis with efficiency rate as

covariate.

**Table S5.** Overview results of whole-brain parametric modulation.

| **Anatomical location** | **Voxels** | **z Value** | **MNI coordinates  (x, y, z)** | **Laterality** |  |
| --- | --- | --- | --- | --- | --- |
| Cuneus (V1) | 104 | 4.104644 | 21, -97, 10 | Right |  |
| Cuneus |  | 3.362528 | 30, -82, 19 | Right |  |
| Lingual gyrus |  | 3.266797 | 21, -88, -2 | Right |  |
| Superior parietal lobule | 99 | 3.877598 | -24, -61, 52 | Left |  |
| Inferior parietal lobule |  | 3.484674 | -33, -52, 46 | Left |  |
| Inferior parietal lobule |  | 3.336889 | -27, -55, 37 | Left |  |
| Visual cortex V4 | 103 | 3.786032 | -21, -82, -2 | Left |  |
| Middle occipital gyrus |  | 3.517043 | -39, -85, 16 | Left |  |
| Middle occipital gyrus |  | 3.42762 | -30, -94, 10 | Left |  |
|  |  |  |  |  |  |

# References

Stolk, A., Noordzij, M. L., Volman, I., Verhagen, L., Overeem, S., van Elswijk, G., Bloem, B., Hagoort, P., & Toni, I. (2014). Understanding communicative actions: a repetitive TMS study. *Cortex; a Journal Devoted to the Study of the Nervous System and Behavior*[,](http://paperpile.com/b/858tAS/DvfL) *51*, 25–34.

Koster-Hale, J., & Saxe, R. (2013). Theory of Mind: A Neural Prediction Problem. *Neuron*, *79*(5), 836–848.
